# Effect of *Luteolin* on 11Beta-Hydroxysteroid Dehydrogenase in Rat Liver and Kidney

**DOI:** 10.1155/2015/834124

**Published:** 2015-06-25

**Authors:** Lei Tang, Bin Deng, Lijuan Shi, Binghua Wei, Bin Ren, Xiaohua Fu

**Affiliations:** ^1^Department of Pharmacy, The First Affiliated Hospital, Sun Yat-Sen University, 58 Zhongshan No. 2 Road, Guangzhou, Guangdong 510080, China; ^2^The Fourth Affiliated Hospital of Guangzhou Medical University, Guangzhou, Guangdong 511447, China; ^3^Department of Pharmacy, Guangzhou Xinhai Hospital, Guangzhou, Guangdong 510300, China

## Abstract

11Beta-hydroxysteroid dehydrogenase (11*β*-HSD) enzymes control the glucocorticoid (GC) signaling, which is essential in regulating homeostasis. Our previous study revealed that *Eclipta prostrata* (EP) affected the activity and expression of 11*β*-HSD enzymes which might improve the efficacy and reduce the adverse drug effects of glucocorticoid in patients undergoing combinational therapy. However, it is still unclear which composition of EP plays a major role and how it works. In this paper, we chose *Luteolin* which is one of the main ingredients of EP and evaluated its effect and metabolism in combination with prednisone. The effects of different concentrations of *Luteolin* extract on prednisone/prednisolone metabolism indicated the enzyme activity of 11*β*-HSD, so the production rate (pmol/min per mg protein) of metabolites was used to indicate enzyme activity. Furthermore, we explored the influence of *Luteolin* on gene and protein expressions of 11*β*-HSD I/II in rat liver and kidney tissue. Our results showed that oral administration of *Luteolin* significantly increased the gene and protein expressions of hepatic 11*β*-HSD I and renal 11*β*-HSD II, which may improve the efficacy and reduce the adverse drug effect of glucocorticoid in clinical application. A potential clinical value of *Luteolin* would also be indicated in combination therapy with prednisone for the treatment of nephrotic syndrome.

## 1. Introduction

Glucocorticoids (GCs) are essential in regulating diverse processes in the development and homeostasis in virtually all body tissues [1]. GCs, which include endogenous cortisol and synthetic drugs, regulate the biosynthesis and metabolism of sugar, fat, and protein [2]. As one of the synthetic GCs, prednisone is commonly used for the treatment of immunological diseases, such as rheumatoid arthritis, systemic lupus erythematosus, polymyositis, and nephrotic syndrome [3, 4]. The activities of GCs are controlled locally and systemically by 11*β*-HSD enzymes [5]. For instance, 11*β*-HSD enzymes are responsible for the activation and inactivation of the synthetic GCs drug prednisone, which is very important in enhancing the curative effect and avoiding unwanted reactions of prednisone [6, 7].

There are two subtypes of 11*β*-HSD enzymes: 11*β*-HSD type 1 (11*β*-HSD I), which acts as an oxoreductase-generating enzyme predominantly activating GCs in liver; 11*β*-HSD type 2 (11*β*-HSD II), which impedes binding of GCs to the nonselective mineralocorticoid receptor (MR) in mineralocorticoid target tissues such as kidney and colon [8–10]. 11*β*-HSD I shows predominant activity in liver, where the preferential usage of NADPH for steroid biosynthesis is thought to convert prednisone to prednisolone [10, 11]. In contrast to the 11*β*-HSD I enzyme, 11*β*-HSD II relies solely on its oxidant cosubstrate, acting exclusively as a dehydrogenase to catalyze the conversion of prednisolone to inactive prednisone in kidney [12, 13]. Moreover, 11*β*-HSD II acts as a “gate-keeper” to protect MR from high concentrations of GCs [14–16]. Kidney injury can cause hypertension and hypokalemia as a consequence of impaired inactivation of cortisol (inhibition of 11*β*-HSD2) [15, 17].

In the previous study, we proved that* Eclipta prostrata* (EP) significantly affected the activity and expression of 11*β*-HSD enzymes, which might improve the efficacy and reduce the adverse drug effects of glucocorticoid in patients undergoing combinational therapy [18]. However, to the best of our knowledge, no further investigation has been performed to show which composition of EP plays a major role and how it works.

By analyzing the composition of many batches of commercial available EP, we found that the main constituents of EP include* Luteolin, Luteolin-7-oxygen-glucosidase*,* Apigenin*, and* Apigenin-7-oxygen-glucosidase*. According to the recent research,* Luteolin*, a flavonoid of EP, was found to potently inhibit 20*α*-hydroxysteroid dehydrogenase [19–21], which belonged to 11*β*-HSD family. Therefore, we wonder if coadministration of* Luteolin* could influence 11*β*-HSD enzymes and the clinical effects in combination with glucocorticoid.

In this paper, we chose* Luteolin* to evaluate its effect and metabolism in combination with prednisone. The production rate (pmol/min per mg protein) of metabolites was used to indicate enzyme activity. Furthermore, we explored the influence of* Luteolin* on gene and protein expressions of 11*β*-HSD I/II in rat liver and kidney tissue.

## 2. Materials and Methods

### 2.1. Experimental Animals

Male Sprague-Dawley rats weighed 200–250 g were purchased from the Medical Experimental Animal Center of Guangdong Province. The animals were housed at an ambient temperature of 20–25°C and relative humidity 40–70% with 12 h-light/dark cycle. They are allowed free access to the standard rodent chow and clean water.

This study was performed with the approval of the local ethical committee and all the experiments were performed according to the National Institutes of Health Guide for the Care and Use of Laboratory Animals. 24 rats were randomly divided into 4 groups and orally administrated with vehicle (1% CMC-Na solution) or* Luteolin* (5 mg/kg, 10 mg/kg, or 20 mg/kg daily) for 14 days. On the next morning, the livers and kidneys of each rat were harvested as soon as possible and stored in liquid nitrogen after being washed with ice-cold 0.9% NaCl solution until usage.

### 2.2. Chemicals and Reagents

*Luteolin* (purity > 98%, batch number: XC071225) was purchased from Xi'an Xiaocao Botanical Development Co., Ltd. (Xi'an, China). Sodium carboxymethyl cellulose (CMC-Na), prednisone, prednisolone, and dexamethasone (internal standard for HPLC analysis) were purchased from Sigma-Aldrich Corporation (St. Louis, MO, USA). Methanol of HPLC grade was purchased from TEDIA Company Inc. (Beijing, China). The following items were purchased from the cited commercial sources: anti-11*β*-HSDI (Abcam, Cambridge, UK, dilution 1 : 800), anti-11*β*-HSD II (Abcam, Cambridge, UK, dilution 1 : 1000), GAPDH (Santa Cruz Biotechnology Inc., Santa Cruz, CA, USA, dilution 1 : 500) antibodies, goat anti-rabbit IgG (HþL) HRP (BS13278), GAPDH (AP0063), and goat anti-mouse IgG (HþL) HRP (BS12478). All other reagents were of analytical grade.

### 2.3. Development and Validation of an HPLC Method

#### 2.3.1. Equipment and Chromatographic Conditions

The equipment employed for HPLC analysis was a Waters system (Waters, Avondale, CA) equipped with a Waters 600 quaternary pump, a Waters 2489 UV-vis detector, a Waters 717 Plus automatic injector and a Workstation (Empower). Chromatographic separation of the analytes was carried out at 40°C by using a Nucleodur 100-5 C18 (5 *μ*m, I.D 4.6 mm × 250 mm), with a mobile phase containing a mixture of A (methanol : 0.2% phosphoric acid = 55 : 45 v/v) and B (acetonitrile). The flow-rate was 1.0 mL/min and absorbance was measured at 240 nm. The total running time was 35 min for each sample.

#### 2.3.2. Method Validation

According to the SFDA guideline, the HPLC method was validated for the specificity of the detection method, the linearity of the calibration curve, accuracy, precision, and recovery. There was no significant interference at the expected retention times of the analytes and interior label. The analytes showed satisfactory linearity over the studied concentration ranges in rat tissues. The intra- and interbatch precision and accuracy were within recommended limits. Therefore, the validated HPLC method was applicable for detecting the concentrations of prednisone and prednisolone.

### 2.4. Preparation of Rat Liver and Kidney Microsomes

Rats were euthanized with diethyl ether before livers and kidneys were harvested and washed with ice-cold 0.9% NaCl solution. The weighted tissues were divided into several parts and homogenized in 2 volumes sucrose homogenization buffer after being washed with ice-cold sucrose at 4°C. The homogenate was subjected to centrifugation at 16000 g for 20 min at 4°C. The supernatant fraction was subjected to ultracentrifugation at 100000 g for 60 min at 4°C. Then the supernatant was discarded and the sediment was washed and resuspended in potassium pyrophosphate buffer and reisolated by ultracentrifugation (100000 g for 60 min at 4°C), successively. The washed microsomes were resuspended in 2 volumes of Tris-HCl buffer containing 20% of glycerol and stored at 80°C until usage.

### 2.5. Microsomal Incubation

In order to obtain the optimal condition of the metabolism of prednisone/prednisolone in liver and kidney, the effects of incubation time and microsome concentration on the metabolism of prednisone in rat liver and kidney microsome were investigated. The method of liver microsomes incubation system was as follows: 20 *μ*L liver microsomes (5 *μ*g/*μ*L), 20 *μ*L standard prednisone (1 mg/mL), and 140 *μ*L potassium phosphate buffer (100 mM, pH 7.4) were incubated for 5 min at 37°C. The reaction was started by adding 20 *μ*L of NADPH and stopped by adding 600 *μ*L methanol after 25 min. Then 10 *μ*L dexamethasone (10 mg/mL, internal standard) and 600 *μ*L methanols were added to 200 *μ*L of the above mixture, vortexed for 1 min, and centrifuged at 10800 rpm for 5 min. The supernatant fraction was obtained and 10 *μ*L of which was injected into HPLC system for analysis. The production rate (pmol/mg protein/min) of metabolites was used to evaluate the enzyme activity.

The method of kidney microsomes incubation system was the same as that of liver except for the concentration of microsomes (3 *μ*g/*μ*L), the promoter agent (NADP+), and the reaction time (45 min).

### 2.6. RT-qPCR

For mRNA quantification, total RNA was extracted with ice-cold Trizol reagent (Sigma) and then reverse-transcribed to complementary DNA (cDNA) using Prime script RT reagent kit according to the manufacturer's protocol. Equal amounts of cDNA were used in real-time quantitative polymerase chain reaction (RT-qPCR). All the PCR reactions were carried out using SYBR Premix Ex TaqTM kit (Takara, Kyoto, Japan) and followed manufacturer's instructions. The 11*β*-HSD I and 11*β*-HSD II expression were measured by fluorescence quantitative RT-qPCR method. The *β*-actin gene was used as an internal control for normalization in parallel with each gene examined. The values obtained for the target gene expression were normalized to *β*-actin and quantified relative to the expression in control samples. The cDNA synthesis conditions were 37°C 15 min for reverse transcription reaction and 85°C 5 s for inactivation of the reverse transcription enzyme, then cooled to room temperature, and stored at −20°C. Amplification was performed with the Light Cycler 2.0 Real-Time Detection System (Roche, Hercules, CA, USA) using the following protocol: 40 cycles (5 s at 95°C and 34 s at 60°C) after an initial activation step for 30 s at 95°C. Primer sequences were shown in Table 1.

### 2.7. Western Blot

The Western blot was carried out to measure the expression of 11*β*-HSD I and 11*β*-HSD II in liver and kidney. Livers and kidneys were excised immediately after the rats were euthanized. Approximately 100 mg of tissues was homogenized in 1 mL of RIPA buffer and 10 *μ*L of phenylmethylsulfonyl fluoride (PMSF). Total protein was extracted by centrifugation at 15000 rpm for 30 min at 4°C and quantified by enhanced bicinchoninic acid (BCA) protein assay kit. An equal amount of protein was resolved by 10% SDS-PAGE and transferred electrophoretically to polyvinylidene difluoride microporous membranes (Millipore, Bedford, MA, USA). The membranes were blocked with 5% skim milk or BSA in TBST buffer (20 mM Tris-HCl, pH 7.6, 140 mM NaCl, and 0.1% Tween-20) for 2 h at 25°C and then subsequently incubated with primary and secondary antibodies. The antibody reactivity was detected by ECL and then quantified by densitometry with ImageJ 1.44p software.

### 2.8. Statistical Analysis

Statistical analyses were performed using SPSS 17.0 statistical software (SPSS Inc., Chicago, USA) statistical software. Results were expressed as the mean ± SD. Student's *t*-test was performed for statistical comparison of the results of enzyme, and the criterion of significance was set at *P* < 0.05. Using one-way analysis of variance (ANOVA), the differences among groups of gene and protein expression were evaluated and considered statistically significant when *P* < 0.05.

## 3. Results

### 3.1. Effects of* Luteolin* on Enzyme Activity of 11*β*-HSD I and 11*β*-HSD II in Rat Liver and Kidney

**Figure 1:**
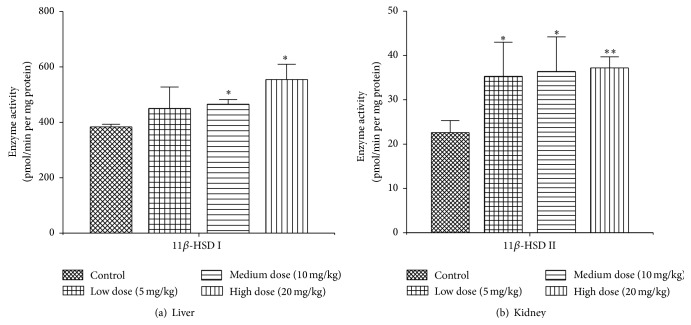
Effects of* Luteolin* on the enzyme activity of 11*β*-HSD I in liver (a) and 11*β*-HSD II in kidney (b). Rats were orally administrated with vehicle (CMC-Na) or* Luteolin* (5 mg/kg, 10 mg/kg, 20 mg/kg) for 14 days. Liver and kidney microsomes were isolated and then 11*β*-HSD I and 11*β*-HSD II enzyme activities were analyzed. ^*∗*^ *P* < 0.05 compared with the control group, ^*∗∗*^ *P* < 0.01 compared with the control group (mean ± SD, *n* = 6).

Effects of* Luteolin* on enzyme activity of 11*β*-HSD I/II in rat liver and kidney were shown in Figure 1. Compared with the negative control group, the 11*β*-HSD I activities from all orally administrated* Luteolin* groups were increased with the medium and high dose groups exhibiting significantly statistical differences (*P* < 0.05). The activities of renal 11*β*-HSD II from all treatment groups were significantly increased compared to the negative control group. The relative 11*β*-HSD II activities of low, medium, and high dose groups were 153.9 ± 33.3%, 155.9 ± 33.9%, and 164.5 ± 10.9%, respectively. There were significant differences (*P* < 0.05) between treatment groups and the negative control group. Therefore,* Luteolin* could significantly induce the activity of hepatic 11*β*-HSD I and renal 11*β*-HSD II, and the effects were positively related to concentration of* Luteolin*. The effects of* Luteolin* on rat liver/kidney 11*β*-HSD indicated that* Luteolin* could affect the metabolism of prednisone/prednisolone and regulate the local tissue concentrations by 11*β*-HSD modulation.

### 3.2. Effects of* Luteolin* on Gene Expression of 11*β*-HSD I and 11*β*-HSD II in Rat Liver and Kidney

**Figure 2:**
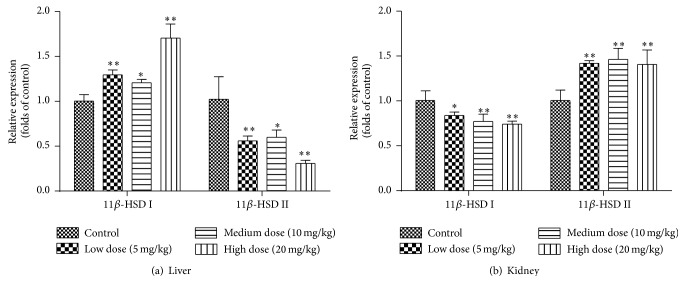
Effects of* Luteolin* on the gene expression of 11*β*-HSD I and 11*β*-HSD II in rat liver (a) and (b). Rats were orally administrated with vehicle (CMC-Na) or* Luteolin* (5 mg/kg, 10 mg/kg, or 20 mg/kg) for 14 days. Liver and kidney were harvested and then 11*β*-HSD I and 11*β*-HSD II mRNA were analyzed. Data are expressed as fold change over the control group (mean ± SD, *n* = 6) ^*∗*^ *P* < 0.05, ^*∗∗*^ *P* < 0.01.

Figure 2 showed the relative gene expressions of 11*β*-HSD I/II in rat liver and kidney after orally administration of vehicle (1% CMC-Na solution) or* Luteolin* at the dosages of 5 mg/kg, 10 mg/kg, or 20 mg/kg daily for 14 days. Compared with the control group, treatment of* Luteolin* (5, 10, and 20 mg/kg) significantly increased hepatic 11*β*-HSD I gene expression by 29%, 20%, and 70%, respectively, whereas those of hepatic 11*β*-HSD II gene expressions were decreased by 45%, 41%, and 70%, respectively. It was found that* Luteolin* significantly decreased the gene expression level of 11*β*-HSD I (*P* < 0.05) and upregulated that of 11*β*-HSD II (*P* < 0.01) in kidney. Therefore,* Luteolin* significantly induced gene expression of hepatic 11*β*-HSD I and renal 11*β*-HSD II, whereas inhibited the gene expression of hepatic 11*β*-HSD II and renal 11*β*-HSD I.

### 3.3. Effects of* Luteolin* on Protein Expression of 11*β*-HSD I and 11*β*-HSD II in Rat Liver and Kidney

**Figure 3:**
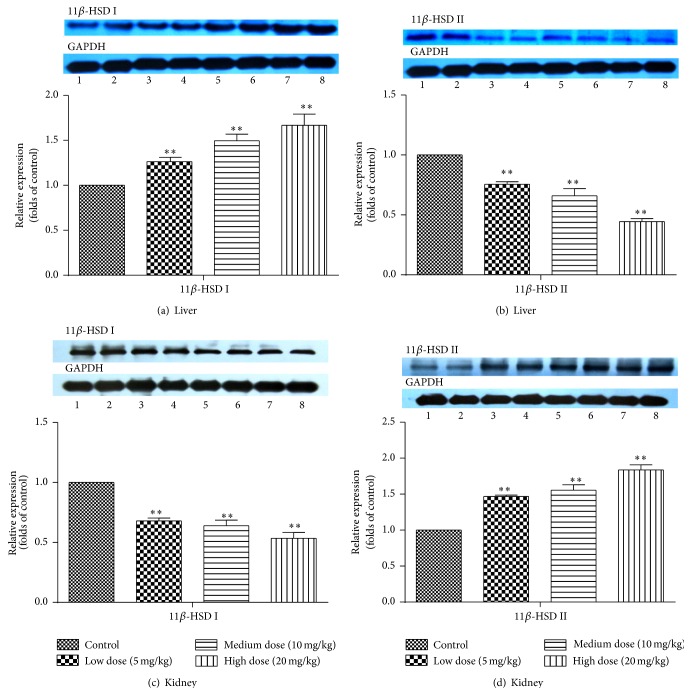
Effects of* Luteolin* on the protein expression of 11*β*-HSD I and 11*β*-HSD II in rat liver (a and b) and kidney (c and d). Rats were orally administrated with vehicle (CMC-Na) or* Luteolin* (5 mg/kg, 10 mg/kg, or 20 mg/kg) for 14 days. Liver and kidney were harvested and then 11*β*-HSD I and 11*β*-HSD II protein levels were analyzed. Data are expressed as fold change over the control group (mean ± SD, *n* = 6) ^*∗*^ *P* < 0.05, ^*∗∗*^ *P* < 0.01. 1, 2: control group; 3, 4:* Luteolin* 5 mg/kg; 5, 6:* Luteolin *10 mg/kg; 7, 8:* Luteolin* 20 mg/kg.

As shown in Figure 3, the relative protein expression levels of 11*β*-HSD I/II in rat liver and kidney were investigated by western blot analysis. Compared with control group, the protein expressions of 11*β*-HSD I in rat liver treated with* Luteolin* (5, 10, and 20 mg/kg) were increased by 26%, 49%, and 66%, respectively (Figure 3(a)), whereas the protein expressions of 11*β*-HSD II were reduced by 24%, 34%, and 56%, respectively (Figure 3(b)). In contrast, the protein expressions of 11*β*-HSD II in rat kidney treated with* Luteolin* (5, 10, and 20 mg/kg) were increased by 47%, 55%, and 84%, respectively, while those of 11*β*-HSD I were decreased by 32%, 37%, and 47%, respectively (Figures 3(c) and 3(d)). The influence of* Luteolin* on protein expression of 11*β*-HSD showed dose-dependent relationship, and high-dose group had the most significant effect. Therefore,* Luteolin* upregulated the protein expression of 11*β*-HSD I and downregulated the protein expression of 11*β*-HSD II in liver. The effects of* Luteolin* on the protein expression of 11*β*-HSD II and 11*β*-HSD I in kidney were opposite compared with those in liver.

## 4. Discussion

In recent studies, a lot of parameters were calculated to represent the enzyme activity of 11*β*-HSD, including measuring the AUC ratio of prednisone/prednisolone in plasma [22–24] and ratio of (THF+5*α*-THF)/THE [15, 25, 26] (THB+5*α*-THB)/THA [27] (THF: tetrahydrocortisol, 5*α*-THF: 5alpha-tetrahydrocortisol, THE: tetrahydrocortisone, THB: tetrahydrocorticosterone, 5*α*-THB: 5alpha-tetrahydrocorticosterone, and THA: 11-dehydro-tetrahydrocorticosterone). Furthermore, 11*β*-HSD activities could also be detected by microsomal incubation with radiolabeled 3H-corticosterone [28, 29] and 3H-cortisone [30] as substrate. The purpose of this study was to examine the effect of drug combination with* Luteolin* on metabolism of prednisone/prednisolone through 11*β*-HSD in liver and kidney. Therefore, prednisone and prednisolone were used as the substrates to estimate the enzyme activity of 11*β*-HSD I/II in the microsomal incubation.

To ensure the accuracy of enzyme activity, the concentration of substrate must exceed enzyme catalysis capacity. Thus, 100 *μ*g/mL prednisone/prednisolone was used which fully met the requirements. Through the comparison and analysis of dissolving in 1%, 3%, 5%, 7%, 10%, 15%, and 20% methyl alcohol, the substrates were dissolved in 10% methyl alcohol because of good solubility.

It has been confirmed that activation of prednisone in patients and animal models improved the nephrotic syndrome; thus prednisolone plasma concentration was lower than normal, which would affect the efficacy of hormone. Meanwhile the activity of 11*β*-HSD II in kidney decreased and could not effectively inactivate glucocorticoids, which would cause water-sodium retention, hypertension, and other mineral corticoid-like adverse reactions.* Luteolin* can induce activity of 11*β*-HSD I in liver, which in turn promote the metabolic activation of prednisone, increase the prednisolone plasma concentration, and improve hormone efficacy. Furthermore,* Luteolin* can accelerate the inactivation of prednisolone in kidney by inducing renal 11*β*-HSD II and thus can weaken the mineralocorticoid hormone-like prednisolone-induced adverse reactions caused by prednisolone. Therefore, the combination of* Luteolin* and prednisone in the treatment of kidney disease could accelerate the inactivation of prednisolone and reduce the adverse effects of hormones. The effect of* Luteolin* on 11*β*-HSD activities in rat liver/kidney microsomes was similar to that of EP, indicating a potential clinical value of* Luteolin* in combination with prednisone for the treatment of nephrotic syndrome.

As shown in Figure 3(a),* Luteolin* induced the gene expression of 11*β*-HSD I and inhibited the gene expression of 11*β*-HSD II in liver. While in Figure 3(b), the gene expression of 11*β*-HSD I was restrained. In contrast, the gene expression of 11*β*-HSD II was induced. It indicates that* Luteolin* can promote the activation and metabolism of prednisone in liver by inducing the gene expression of 11*β*-HSD I. Meanwhile, the upregulated gene expression of 11*β*-HSD II can accelerate the inactivation of prednisolone in kidney and reduced the adverse effects.

Compared with the control group, continuous administration of* Luteolin* for 14 days significantly upregulated protein expressions of 11*β*-HSD I and downregulated levels of 11*β*-HSD II in liver, while* Luteolin* downregulated the expression of 11*β*-HSD I and upregulated the expression of 11*β*-HSD II in kidney. The effect of* Luteolin* on 11*β*-HSD protein was concentration-dependent, and high-dose group had the most significant effect. The results of RT-qPCR, western blot, and in vitro activity showed that* Luteolin* could affect the interconversion between prednisolone and prednisone by inducing mRNA and protein expression of hepatic 11*β*-HSD I and renal 11*β*-HSD II. The results suggest that the drug interactions between* Luteolin* and glucocorticoids should be paid attention to, which will provide a theoretical basis for clinical combination therapy.

The content of* Luteolin* in EP was 0.45 mg/g, determined by HPLC. The content of* Luteolin* was significantly higher than the other flavonoids. In the previous research, oral administration of EP significantly increased the activity and expression of 11*β*-HSD I in the liver and 11*β*-HSD II in the kidney which was similar with* Luteolin*. Therefore, conclusion could be drawn that* Luteolin* is one of the constituents that is responsible for the clinical effects of EP.

## 5. Conclusion

In summary, oral administration of* Luteolin* significantly increased the gene and protein expression of hepatic 11*β*-HSD I and renal 11*β*-HSD II, which may improve the efficacy and reduces the adverse drug effects of glucocorticoid in clinical application. Our study addressed a potential clinical value of* Luteolin* in combination with prednisone in the treatment of nephrotic syndrome.

## Figures and Tables

**Table 1:** Primer sequences used for real-time polymerase chain reaction.

Gene	Primer sequences
11*β*-HSD I	
Forward	5′-TGACCAAGGTCAACGTGTCCA-3′
Reverse	5′-ATGATCTCCAGGGCGCATTC-3′
11*β*-HSD II	
Forward	5′-GACCTTAGCCCCGTTGTAGATG-3′
Reverse	5′-GGCAGGTAGTGGTGGATGAAA-3′
*β*-actin	
Forward	5′-GGAGATTACTGCCCTGGCTCCTA-3′
Reverse	5′-GACTCATCGTACTCCTGCTTGCTG-3′
